# Efficacy of photobiomodulation in perineal trauma after vaginal delivery: a protocol for a randomized, blinded clinical trial

**DOI:** 10.1007/s10103-026-05036-7

**Published:** 2026-09-15

**Authors:** Alianny Rodrigues, Magdalena Brilhante, Emilly Lima, Isabelly Regalado, Maria Thereza Micussi, Laiane Eufrásio, Adriana Magalhães

**Affiliations:** 1 Faculdade de Ciências da Saúde do Trairi, Universidade Federal do Rio Grande do Norte, Santa Cruz, Brazil; 2 Departamento de Fisioterapia, Universidade Federal do Rio Grande do Norte, Natal, Brazil

**Keywords:** Laser, Pain, Postpartum period, Postpartum women, Vaginal delivery, Perineal trauma.hi

## Abstract

**Objective:**

To evaluate the efficacy of photobiomodulation (PBM) in reducing perineal pain and obstetric vulvar edema, as well os its effects on maternal functioning during the immediate postpartum period.

**Methods:**

This randomized, controlled, double-blind clinical trial protocol will include women aged over 15 years, within 12 h after vaginal delivery, presenting with first- or second-degree perineal trauma and/or vulvar edema and pain ≥ 3 on the Numeric Rating Scale. Participants will be randomized in blocks of four to receive PBM or a sham intervention alongside usual postpartum care. A single PBM session will be performed using 808-nm infrared laser light at an output power of 100 mW. Assessments will be performed at baseline, immediately after the intervention, 12 h, 40 days, and three months postpartum. Outcomes will include pain, pressure pain threshold, vulvar edema area, maternal functioning, treatment satisfaction, urinary incontinence, and sexual function. Statistical analysis will follow the intention-to-treat principle using linear mixed-effects models, with group, time, and group × time interaction as fixed effects and participant as a random effect. Post-hoc comparisons will use Bonferroni correction, with a significance level of 5%.

**Conclusion:**

PBM is expected to reduce perineal pain and vulvar edema and improve maternal functioning. The randomized design, sham control, blinded outcome assessment, and longitudinal analysis are expected to provide evidence regarding the efficacy and safety of PBM as a non-pharmacological strategy for postpartum care.

**Trial Registration:**

Brazilian Clinical Trials Registry (REBEC), RBR9sgdsm2, approved on July 7, 2026.

## Introduction

The pelvic floor (PF) is a complex structure composed of muscles, ligaments, and fascia that closes the inferior pelvic outlet and supports the pelvic organs. During pregnancy and vaginal birth, this structure is subjected to substantial biomechanical stress, predisposing women to pelvic floor disorders (PFDs) during the postpartum period [1]. 

Approximately 85% of women undergoing vaginal birth experience some degree of perineal trauma, including spontaneous perineal tears and/or episiotomy. Perineal tears are classified into four degrees according to the Royal College of Obstetricians and Gynaecologists and the American College of Obstetricians and Gynecologists. First- and second-degree tears are considered minor injuries, involving superficial tissues and the perineal muscles, whereas third- and fourth-degree tears are classified as severe because they involve the anal sphincter complex and the rectal mucosa [2]. 

In the short term, perineal trauma may result in pain, bleeding, wound complications, infection, and prolonged hospitalization. In the long term, it may lead to persistent pain, dyspareunia, urinary and fecal incontinence, fistula formation, and pelvic organ prolapse. [3–4] Severe perineal lacerations, particularly third- and fourth-degree tears, may substantially affect women’s quality of life and mental well-being, highlighting the importance of identifying risk factors and implementing preventive strategies during childbirth.4 In addition to these physical consequences, these conditions may adversely affect maternal functioning, quality of life, emotional well-being, and maternal–infant bonding during the postpartum period. [4–5]

Perineal trauma may also be associated with vulvar edema, a condition related to pregnancy-specific factors such as preeclampsia, diabetes, prolonged labor, operative vaginal delivery, hypoproteinemia, and anemia [6, 7]. Although its pathophysiology has not been fully elucidated, it is believed to involve alterations in hydrostatic and oncotic pressures, as well as the characteristics of the loose connective tissue of the vulva. Current treatment options are limited and predominantly pharmacological, including corticosteroids, hypertonic saline compresses, albumin, low-molecular-weight heparin, and antibiotics [8–10]. 

Photobiomodulation (PBM) has emerged as a promising non-pharmacological intervention because of its analgesic, anti-inflammatory, and anti-edematous effects. By stimulating mitochondrial chromophores, PBM enhances adenosine triphosphate production and modulates important biological processes, including inflammation, angiogenesis, lymphangiogenesis, and the release of endogenous opioids [11–13]. Although the body of evidence supporting PBM for pain management is growing, robust randomized controlled trials evaluating its effects on postpartum perineal pain and obstetric vulvar edema remain scarce.

Accordingly, this study protocol aims to describe a randomized clinical trial designed to evaluate the efficacy of photobiomodulation in reducing perineal pain during the postpartum period (change from baseline). Secondary objectives include evaluating vulvar edema, maternal functioning, urinary incontinence, sexual function, and patient satisfaction.

## Methods

### Study design

This study is a randomized, controlled, parallel-group, superiority, double-blind clinical trial designed to evaluate the effects of photobiomodulation (PBM) on perineal pain during the immediate postpartum period.

### Participants

The study population will consist of women aged over 15 years in the immediate postpartum period following vaginal birth who present with perineal trauma and/or obstetric vulvar edema and are admitted to the Pre-labor, Labor and Postpartum Unit, the Normal Birth Center, or the Surgical Ward of University Hospital. Eligible participants who provide written informed consent will be randomly assigned to one of the study groups.

#### Inclusion criteria

Women will be eligible if they meet all of the following criteria: age > 15 years; pain intensity ≥ 3 on the Numeric Rating Scale (NRS); first- and/or second-degree perineal trauma and/or vulvar edema; immediate postpartum period (up to 12 h afterchildbirth) (Table 1).

**Table 1:** Participant timeline: Schedule of enrollment, interventions, and assessments

	Trial period			
Timepointb	Enrollment	Post-randomization		Close-out
	July 2025 to December 2026 T0	Post-immediate postpartum T1	after 12 h T2	40 days, and 3 monthst T3,T4
ENROLLMENT:				
Eligibility screen	x			
Informed consent	x			
Randomization				
INTERVENTION/ COMPARATOR:				
G1 – Photobiomodulation	x			
G2 – Sham PBM + Usual care				
ASSESSMENTS:		x		
Pain (NRS)	x			
Vulvar edema	x	x	x	
Immediate postpartum functioning	x	x	x	
Patient satisfaction	x	x	x	
Patient Global Impression of Change (PGIC)	x	x	x	
ICIQ-SF				x
WHODAS 2.0				x
FSFI				x
Adverse events	x	x	x	x

Abbreviations *PBM* photobiomodulation, *T0* pre-intervention, *T1* immediately after the intervention, *T2* 12 hours after the intervention, *T3* 40 days postpartum, *T4* 3 months postpartum, *NRS* Numeric Rating Scale, *WHODAS 2.0* World Health Organization Disability Assessment Schedule 2.0, *ICIQ-SF* International Consultation on Incontinence Questionnaire–Short Form, *FSFI* Female Sexual Function Index

#### Exclusion criteria

Women will be excluded if they present with vaginal wounds unrelated to perineal trauma, clinical signs or a confirmed diagnosis of vaginal infection, vulvar hematoma, cognitive impairment, or communication difficulties that preclude participation.

Eligible participants will be enrolled and randomized into one of two groups: photobiomodulation (G1) or sham photobiomodulation plus usual care (G2). The intervention will be delivered by physiotherapists with at least one year of clinical experience in pelvic floor physiotherapy and previous training in photobiomodulation. All therapists will undergo standardized training before the initiation of the study.

### Sample size calculation

The sample size was calculated using G*Power software (version 3.1.9.7), based on a two-tailed independent-samples Student’s *t* test for the comparison of means between two independent groups. A significance level of 5% (α = 0.05), statistical power of 80% (1 − β = 0.80), and an allocation ratio of 1:1 were assumed. Based on the findings of Alvarenga et al. ^16^. a mean perineal pain score of 4.5 (SD = 3.0) was assumed for the experimental group and 2.0 (SD = 2.2) for the sham group, resulting in an estimated effect size (Cohen’s *d*) of 0.784. The analysis indicated a minimum sample size of 54 participants, with 27 participants per group, achieving an actual statistical power of 80.75%. Considering an anticipated dropout rate of 20%, the sample size was increased to 68 participants, with 34 participants allocated to each group [14–16]. 

### Randomization and allocation concealment

**Fig. 1:**
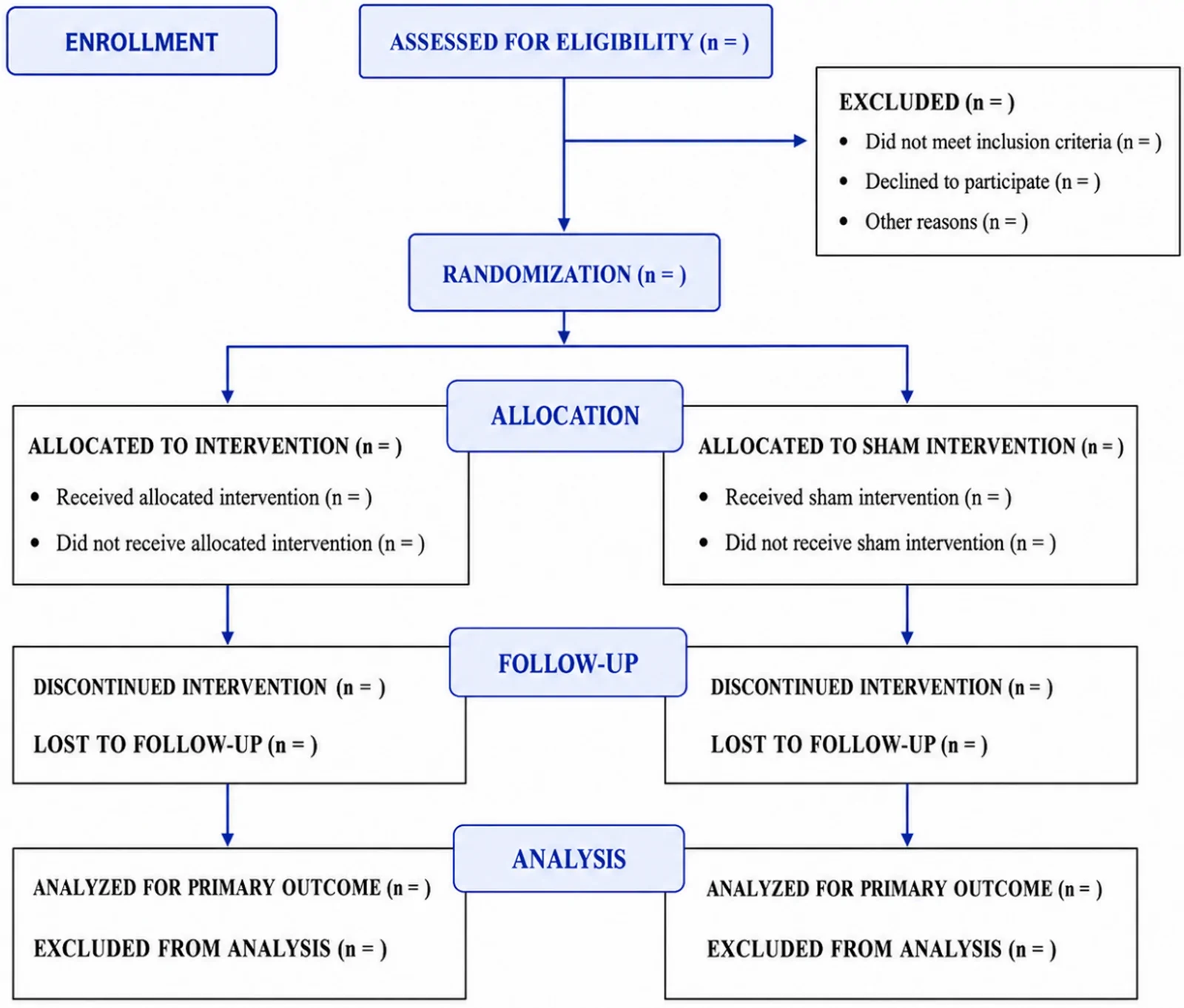
CONSORT diagram of the flow of participants throughout the research process

Participants will be randomized in blocks of four according to a sequence of random numbers ranging from 1 to *x* (the total number of participants to be randomized), generated using Random Allocation Software version 1.0. An independent researcher not involved in participant recruitment, intervention delivery, outcome assessment, or data analysis will generate the randomization sequence and prepare sequentially numbered, opaque, sealed, and stapled envelopes containing the treatment allocation, in accordance with the CONSORT recommendations (Fig. 1) [17].

### Blinding

The therapist responsible for administering the intervention could not be blinded to treatment allocation because knowledge of the device setting was necessary to deliver either active photobiomodulation or sham photobiomodulation according to the study protocol. To minimize potential performance bias, the therapist followed a standardized intervention protocol, with identical patient positioning, application procedures, treatment duration, and interaction with participants in both groups. The sham intervention reproduced the same procedures as the active intervention, except for the absence of therapeutic light emission.

The therapist was not involved in outcome assessment or data analysis. Participants were blinded to treatment allocation through the use of fully occlusive protective goggles, which prevented the perception of light emission. Neither the active nor sham intervention produced a sensation of heat. Outcome assessors and data analysts also remained blinded to group allocation. At the end of the intervention, participants were asked to indicate which treatment they believed they had received, allowing assessment of the success of participant blinding by comparing their perceived allocation with their actual treatment allocation.

Unblinding occurred only in the event of a serious adverse event or when knowledge of treatment allocation was required for clinical decision-making and was performed by an independent researcher who was not involved in outcome assessment or data analysis.

### Intervention

The study will comprise two groups: (G1) photobiomodulation (PBM) and (G2) sham PBM, with both groups receiving usual postpartum care. Women diagnosed with first- and/or second-degree perineal trauma and/or obstetric vulvar edema will be invited to participate and randomly assigned to one of the two study groups.

A single treatment session will be performed during the immediate postpartum period, within 12 h after childbirth, with an approximate duration of 20 min. Before the intervention, participants will undergo the baseline assessment, including evaluation of perineal pain using the Numeric Rating Scale (NRS). In both groups, participants will receive individualized care and standardized education regarding pelvic floor anatomy, perineal trauma, vulvar edema, and perineal care. At the end of the immediate postpartum assessment, all participants will receive an educational leaflet containing recommendations for perineal care.

Participants allocated to G1 will receive PBM using the TERAPY-EC^®^ device with a wavelength of 808 nm (infrared), output power of 100 mW, beam area of 1 mm², and power density of 1.02 W/cm².

For participants presenting with first- and/or second-degree perineal trauma and pain ≥ 3 on the NRS, infrared light (808 nm) will be applied using a point-by-point technique, with an energy density of 60 J/cm² for 60 s per application point, corresponding to 6 J of energy per point. The irradiation will be performed around the perineal lesion and along the course of the pudendal nerve. One or two irradiation points will be used according to the size and extent of the lesion, with the exact number of points documented for each participant.

For participants presenting with both perineal pain and vulvar edema, the complete perineal pain protocol described above will be performed first. Subsequently, PBM will be applied to the edematous vulvar area using 808-nm infrared light at an energy density of 10 J/cm² for 10 s per point, with a 2-cm distance between adjacent application points. In addition, two irradiation points will be applied to the inguinal region, with 20 J/cm² per point, corresponding to 20 s per point, for a total of 40 J/cm² and 40 s in the inguinal region. The treatment sequence will therefore consist of perineal irradiation first, followed by irradiation of the edematous vulvar area and, finally, the two inguinal points, when vulvar edema is presente (Table 2).

**Table 2:** Photobiomodulation intervention parameters for perineal pain and vulvar edema

Parameter	Perineal pain	Vulvar edema
Clinical condition	First- and/or second-degree perineal trauma + pain ≥ 3 on the NRS	Obstetric vulvar edema
Wavelength	808 nm (infrared)	808 nm (infrared)
Output power	100 mW	100 mW
Beam area	1 mm²	1 mm²
Power density	1.02 W/cm²	1.02 W/cm²
Application technique	Point-by-point	Point-by-point
Energy density	60 J/cm²	10 J/cm²
Application time	60 s per point	10 s per point
Energy per point	6 J	1 J
Number of points	1–2 points, according to lesion size	According to the extent of the edematous area
Application site	Around the lesion and along the course of the pudendal nerve	Over the edematous vulvar area
Distance between points	—	2 cm
Additional application	—	2 points in the inguinal region
Parameter for inguinal points	—	20 J/cm² per point
Application time at inguinal points	—	20 s per point
Energy at inguinal points	—	2 J per point
Total energy at inguinal points	—	4 J
Treatment sequence	Application around the lesion and along the pudendal nerve pathway	Edematous area → inguinal region

Note: For participants presenting with both perineal pain and vulvar edema, the perineal pain protocol will be performed first, followed by application to the edematous area and, subsequently, to the two inguinais points

Participants allocated to G2 will undergo the same application procedure, positioning, treatment sequence, and approximate treatment duration as participants in G1. However, the device will remain inactive, with no light emission, thereby providing a sham intervention. The same device and application sites will be used to maintain participant blinding. Participants in the sham group will also receive the same standardized postpartum education and usual care provided to the PBM group.

All intervention procedures will be performed by trained physiotherapists following a standardized operating procedure (SOP). The parameters, number and location of application points, duration, and energy delivered will be recorded for each participant to ensure treatment fidelity and reproducibility.

### Intervention modification and discontinuation

The intervention will be discontinued if participants report intolerable pain exceeding their pre-intervention Numeric Rating Scale (NRS) score, increased local tenderness accompanied by a sharp or stabbing sensation in the treated area, or any adverse event that may compromise participant safety. Participants may also withdraw from the study at any time without providing a reason. All withdrawals and their respective reasons will be documented and reported.

### Intervention adherence

Adherence will be monitored through attendance records and completion of all scheduled treatment sessions. A standardized checklist will be used to ensure protocol fidelity during each intervention session. Any protocol deviations will be documented. Because the intervention will be administered during hospitalization, an adherence rate of 90% is expected.

### Concomitant care

All participants will receive standard postpartum care, including guidance on perineal hygiene, posture, hydration, and activity modification. The use of analgesic medications will be permitted when clinically indicated and will be recorded in both groups to account for potential confounding factors.

### Recruitment

Participants will be recruited from the Pre-labor, Labor and Postpartum Unit (PPP), the Normal Birth Center (CPN), and the Surgical Ward of University Hospital. Recruitment will be conducted daily through active screening of medical records and direct contact by trained researchers.

### Participant characteristics and outcome assessments

Participants will undergo five assessments: three during the immediate postpartum period (baseline, immediately after the intervention, and 12 h after the intervention), one at 40 days postpartum, and one at 3 months postpartum. The schedule of assessments and assessment instruments is summarized in Table 3.

**Table 3:** Detailed description of study outcomes

Outcome	Instrument/test	Description of the instrument/test	Scoring	Assessment timepoint	Clinically meaningful effect
Pain	Numeric Rating Scale (NRS)	A unidimensional instrument widely used to assess self-reported pain intensity.	0 (no pain) to 10 (worst pain imaginable).	T0, T1, and T2	Reduction of ≥ 2 points on the NRS.
	Digital pressure algometer	Assesses the pressure pain threshold in the bilateral peritraumatic region by applying gradually increasing pressure.	Value recorded in kgf/cm².	T0, T1, and T2	Increase in the pressure pain threshold.
Vulvar edema	Standardized photography and measuring tape	Objective assessment of vulvar edema using standardized photographic documentation and measurement of the edematous area.	Measurement in millimeters and photographic analysis.	T0, T1, and T2	Reduction in vulvar edema size.
Immediate postpartum functioning	Likert scale	Assesses difficulty in performing functional activities during the immediate postpartum period (standing up, sitting down, walking, and caring for the newborn).	1 = no difficulty to 5 = extreme difficulty.	T0, T1, and T2	Reduction in functional difficulty.
Patient satisfaction	Visual Analog Scale (VAS)	Assesses participant satisfaction with the treatment received.	0 = no satisfaction to 10 = maximum satisfaction.	T0 and T3	Increased satisfaction compared with the baseline assessment.
Global perceived change	Patient Global Impression of Change (PGIC)	Assesses the participant’s subjective perception of clinical improvement following the intervention.	1 = no change to 7 = very much improved.	T1 and T2	Score ≥ 5, indicating perceived improvement.
Late and remote postpartum functioning	World Health Organization Disability Assessment Schedule 2.0 (WHODAS 2.0)	Assesses functioning across six domains of daily life: cognition, mobility, self-care, getting along with others, life activities, and participation.	1 = no difficulty to 5 = extreme difficulty.	T3 and T4	Reduction in the total WHODAS 2.0 score.
Urinary incontinence	International Consultation on Incontinence Questionnaire–Short Form (ICIQ-SF)	Assesses the frequency and amount of urinary leakage and its impact on quality of life.	Total score ranging from 0 to 21.	T3 and T4	Reduction of approximately 2.5–5 points; changes of ≥ 4 points represent a clinically important improvement.
Sexual function	Female Sexual Function Index (FSFI)	Assesses sexual desire, arousal, lubrication, orgasm, satisfaction, and pain during the previous four weeks.	Total score ranging from 2 to 36; cutoff score = 26.55.	T4	Score > 26.55, indicating preserved sexual function.
Adverse events	Standardized clinical record	Assessment of intervention-related adverse events (increased pain, discomfort, skin irritation, hematoma, allergic reaction, or other complications).	Presence/absence; classified according to type and severity.	T1, T2, T3, and T4	Absence of serious treatment-related adverse events.

Legend *T0* pre-intervention, *T1* immediately after the intervention, *T2* 12 hours after the intervention, *T3* 40 days postpartum (late postpartum period), *T4* 3 months postpartum (remote postpartum period)

The baseline assessment will include the collection of sociodemographic and obstetric data, followed by a standardized physical examination. Perineal pain intensity will be assessed using the Numeric Rating Scale (NRS) and pressure algometry. The NRS is an 11-point scale ranging from 0 (no pain) to 10 (the worst pain imaginable), with pain categorized as mild (1–3), moderate (4–6), or severe (7–10). Pain intensity will be assessed using standardized verbal descriptors [18]. 

Pressure pain threshold will be measured using a calibrated MED.DOR^®^ digital pressure algometer, with readings expressed in kgf/cm². The device will be applied perpendicularly to the bilateral peritraumatic region, with one measurement obtained on each side. Participants will be instructed to indicate the moment when the sensation of pressure becomes painful by saying “stop,” and this value will be recorded as the pressure pain threshold. To ensure biosafety, the algometer probe will be covered with disposable plastic film and disinfected with 70% alcohol before each assessment [19].

The use of analgesic medication will be recorded at each assessment point, including the type of medication, dose, route of administration, and time elapsed between medication administration and pain assessment.

Vulvar Edema Area the area of vulvar edema will be quantified using digital image analysis with ImageJ software (National Institutes of Health, Bethesda, MD, USA). For each photograph, the edematous region will be manually outlined using either the Freehand Selection or Polygon Selection tool according to the anatomical boundaries of the edema. The software will automatically calculate the area of the region of interest (ROI). Images will be calibrated using a reference scale positioned in the same plane as the photographed area, allowing measurements to be expressed in square millimeters (mm²).

Participant satisfaction with the intervention will be assessed using a Visual Analog Scale (VAS) for satisfaction, ranging from 0 (no satisfaction) to 10 (maximum perceived satisfaction), administered at the final assessment [20]. 

Global perceived change will be assessed using the Global Perceived Effect Scale (GPE), which consists of seven response categories ranging from 1 (no change) to 7 (very much better). This outcome will be assessed immediately after the intervention and again 12 h later [21]. 

Immediate postpartum functioning will be evaluated using a 5-point Likert scale, assessing the level of difficulty in performing activities such as standing up, sitting down, walking, and caring for the newborn.

During the late and remote postpartum follow-up assessments, functioning will be evaluated using the World Health Organization Disability Assessment Schedule 2.0 (WHODAS 2.0), which assesses six domains: cognition, mobility, self-care, getting along with others, life activities, and participation. Responses are scored on a 5-point scale ranging from 1 (no difficulty) to 5 (extreme difficulty) [22]. 

Urinary incontinence will be investigated based on participants’ self-reported symptoms and the International Consultation on Incontinence Questionnaire–Short Form (ICIQ-SF), a validated instrument that evaluates the frequency, severity, and impact of urinary leakage on quality of life. Total scores range from 0 to 21, with higher scores indicating greater symptom severity and impact.

Sexual function will be assessed at 3 months postpartum using the Female Sexual Function Index (FSFI), which comprises 19 items distributed across the domains of desire, arousal, lubrication, orgasm, satisfaction, and pain. Total scores range from 2 to 36, with a cutoff score of ≤ 26.55 indicating possible female sexual dysfunction.

All study outcomes will be assessed by previously trained examiners who will remain blinded to participant allocation. The assessment instruments used in this study (NRS, pressure algometry, VAS for satisfaction, Global Perceived Effect Scale, WHODAS 2.0, ICIQ-SF, and FSFI) have demonstrated adequate psychometric properties, including validity and reliability, and are widely used in clinical research involving women during pregnancy and the postpartum period. 

### Patient safety

Participant autonomy will be fully respected, and participants may withdraw from the study at any time without any consequences. Discontinuation criteria include refusal to continue the assessments or the intervention.

Potential risks include mild discomfort during the assessments, transient pain during pressure algometry, and a minimal risk of infection. These risks will be minimized through standardized procedures, private assessment settings, and rigorous equipment disinfection protocols.

In the event of an adverse event, the intervention will be discontinued immediately. All participants will have access to comprehensive medical care at University Hospital, including legally required compensation, if applicable.

### Expected outcomes

Photobiomodulation is expected to significantly reduce perineal pain and vulvar edema during the immediate postpartum period, achieving both statistical and clinical significance. Improvements are also expected in sexual function, maternal functioning, patient satisfaction, urinary incontinence, and quality of life.

If confirmed, these findings may support photobiomodulation as an effective, safe, and clinically relevant intervention for postpartum care, with positive effects on postpartum recovery and sexual health.

### Data management

To ensure confidentiality and minimize potential discomfort, participants will be identified using coded abbreviations rather than their names. Interviews and assessments will be conducted in a private setting with restricted access, and participants will have the option of being accompanied during the procedures.

All data will be recorded in standardized Case Report Forms (CRFs) by trained researchers. Data entry will be performed independently by two researchers using a double-entry system to ensure accuracy. Following data entry, the dataset will be checked for inconsistencies and validated before analysis. A coding system will be applied to all variables, and a data dictionary will be developed to standardize data management procedures.

Assessment forms and image records will be stored securely, with access restricted to the principal investigator. Electronic data will be password-protected and stored on secure institutional servers. All research materials will be retained for five years after study completion and subsequently destroyed, including the permanent deletion of digital files.

### Participant retention and follow-up

Strategies to maximize participant retention will include clear communication regarding the importance of follow-up assessments, flexible scheduling of follow-up visits, and reminder contacts by telephone or text message.

Participants who fail to attend follow-up assessments will be documented, and the reasons for withdrawal will be recorded whenever possible. All randomized participants will be included in the analyses according to the intention-to-treat principle.

### Statistical analysis

Statistical analyses will be performed using the Statistical Package for the Social Sciences (SPSS), version 20.0 (IBM Corp., Armonk, NY, USA), adopting a significance level of 5% (*p* < 0.05). Descriptive statistics will be presented as absolute and relative frequencies for categorical variables and as means, standard deviations (SD), and 95% confidence intervals (95% CI) for continuous variables.

The primary analysis of longitudinal outcomes will be performed using linear mixed-effects models. Treatment group (photobiomodulation and sham), time (T0: baseline; T1: immediately after the intervention; T2: 12 h after the intervention; T3: 40 days postpartum; and T4: 3 months postpartum), and the group × time interaction will be included as fixed effects, while participant will be included as a random effect to account for the correlation among repeated measurements within the same participant. The group × time interaction will be considered the primary parameter for determining whether changes in outcomes over time differ between the photobiomodulation and sham groups. Baseline outcome values will be included in the model when appropriate.

Model assumptions will be assessed by examining the distribution of residuals and the homogeneity of variance. If substantial deviations from model assumptions are identified, appropriate data transformations or alternative statistical approaches will be considered.

For baseline comparisons, categorical variables will be analyzed using Pearson’s chi-square test or Fisher’s exact test, as appropriate. Continuous variables will be analyzed using the independent-samples Student’s t-test for approximately normally distributed data or the Mann–Whitney U test for non-normally distributed data.

When a significant group × time interaction is identified, post-hoc pairwise comparisons will be performed to determine differences between groups at each assessment time point and changes within each group over time. The Bonferroni correction will be applied to adjust for multiple comparisons and control the family-wise type I error rate.

The magnitude of the intervention effect will be estimated using mean differences (MD) for continuous outcomes and relative risks (RR) for dichotomous outcomes, with corresponding 95% confidence intervals (95% CI). The number needed to treat for benefit (NNTB) will be calculated for dichotomous outcomes when applicable.

All primary analyses will follow the intention-to-treat principle. For longitudinal outcomes analyzed using linear mixed-effects models, missing outcome data will be handled using all available observations under the assumption that data are missing at random (MAR), without imputing individual missing time points in the primary analysis. The pattern and reasons for missing data will be described and compared between treatment groups. If the proportion of missing data is substantial, a sensitivity analysis using multiple imputation will be performed to assess the robustness of the results under different assumptions regarding missing data.

### Data monitoring

A Data Monitoring Committee (DMC) will not be established because of the low-risk nature of the intervention. Study oversight will be conducted by the principal investigator, who is independent of outcome assessment.

### Interim analysis

No interim analyses are planned because of the short duration of the intervention and the minimal risks associated with the study.

### Trial monitoring

The study will be monitored by the principal investigator, who will oversee protocol adherence, data quality, and participant safety. Periodic internal audits will be conducted to verify the accuracy, integrity, and compliance of the data with ethical and methodological standards.

The clinical trial will be coordinated by the principal investigator, who will be responsible for supervising all phases of the study, including participant recruitment, intervention delivery, data management, and statistical analysis.

No formal steering committee or outcome adjudication committee will be established because of the low-risk nature and single-center design of the study. Data management will be performed by trained members of the research team under the supervision of the principal investigator.

### Adverse events

Adverse events related to photobiomodulation are expected to be minimal. Any discomfort, increase in pain, or unexpected reaction occurring during or after treatment will be recorded and monitored. Participants will be actively questioned about adverse events at each assessment. If any risk or complication arises, the intervention will be discontinued immediately, and appropriate clinical care will be provided.

Participants who withdraw because of adverse events will remain included in the analyses according to the intention-to-treat principle.

### Patient and public involvement

The conception of this study was informed by needs identified in clinical practice based on reports from postpartum women regarding pain and functional limitations during the immediate postpartum period. In addition, physiotherapists working at the study site participated in discussions during the planning phase of the research, contributing to the definition of the study outcomes, assessment methods, and proposed intervention. The study findings will subsequently be disseminated to the participants and the healthcare professionals involved.

## Discussion

Considering the potential analgesic, anti-inflammatory, and anti-edematous effects of photobiomodulation, this intervention may represent a useful resource for women experiencing perineal trauma and/or obstetric vulvar edema during the immediate postpartum period. Reduction of perineal pain and vulvar edema may facilitate mobility, sitting, walking, self-care, and activities related to newborn care, thereby contributing to maternal recovery during the first hours after childbirth. In addition, PBM is a non-pharmacological intervention that can be applied locally and may be incorporated into physiotherapeutic postpartum care. Previous studies have demonstrated beneficial effects of photobiomodulation and low-level laser therapy on pain and tissue healing, although evidence specifically addressing postpartum perineal pain and obstetric vulvar edema remains limited [11–13, 15]. 

The immediate postpartum period may represent an important therapeutic window because perineal pain and edema can interfere with maternal functional recovery during the first hours after vaginal birth. In this context, the present study was designed to investigate whether a single application of PBM performed within 12 h after childbirth can provide clinically meaningful improvements in perineal pain and vulvar edema. The intervention was standardized according to wavelength, power, energy density, application time, and anatomical sites, allowing reproducibility and facilitating future clinical application.

The use of a sham intervention associated with usual postpartum care is an important methodological feature of this trial, as it allows the effects of PBM to be distinguished from those of routine care and contextual or placebo-related effects. In addition, participants, outcome assessors, and data analysts will be blinded to treatment allocation, while the intervention procedures will be standardized.

However, this study has some limitations. The evaluation focuses on the effects of a single PBM application during the immediate postpartum period, and therefore the study will not determine whether repeated applications could produce additional or sustained benefits. Although follow-up assessments at 40 days and 3 months will allow the investigation of maternal functioning, urinary incontinence, and sexual function, these outcomes cannot be interpreted as direct evidence of a long-term effect of a single PBM session.

If PBM demonstrates clinically meaningful benefits, its use may represent a feasible non-pharmacological strategy for the management of postpartum perineal pain and vulvar edema, particularly within physiotherapy-based postpartum care. Future studies should investigate repeated applications, different treatment parameters, cost-effectiveness, and longer-term effects on perineal healing, persistent pain, maternal functioning, sexual function, and other postpartum outcomes.

## Conclusion

This protocol describes a randomized, controlled, double-blind clinical trial designed to evaluate the efficacy of photobiomodulation for perineal pain and obstetric vulvar edema in the immediate postpartum period. By combining a standardized PBM protocol with sham treatment, blinded outcome assessment, and clinically relevant postpartum outcomes, the study is expected to contribute evidence to an area in which robust clinical trials remain limited. If its efficacy and safety are demonstrated, PBM may represent a feasible non-pharmacological approach to support postpartum recovery and improve maternal functioning and well-being.

## Protocol amendments

Any important modifications to the study protocol (e.g., eligibility criteria, outcomes, or analytical methods) will be documented and communicated to the relevant stakeholders, including investigators, participants (when applicable), and the Brazilian Clinical Trials Registry (REBEC).

## Post-trial care

Participants will continue to receive standard care after completion of the study. Any adverse events identified during the clinical trial will be monitored until resolution. Participants requiring additional care will be referred to the appropriate clinical services.

### Supporting Information

The SPIRIT 2025 Checklist was used to guide the development and reporting of this study protocol. The completed checklist is provided as supplementary material.

## Data Availability

No datasets were generated or analysed during the current study.
